# Endobronchial leiomyoma; report of a case successfully treated by bronchoscopic resection

**Published:** 2010

**Authors:** Nourieh Sharifi, Seyed Hossein Fattahi Massoum, Mahdi Karimi Shahri, Alireza Rezaei, Amir Ansa Ashari, Alireza Sharifian Attar, Ahmad Amozeshi

**Affiliations:** a Associate Professor of Pathology, Mashhad University of Medical Sciences, Mashhad, Iran; b Professor of Thoracic Surgery, Mashhad University of Medical Sciences, Mashhad, Iran; c Resident of Pathology, Mashhad University of Medical Sciences, Mashhad, Iran; d Assistant Professor of Internal Medicine, Mashhad University of Medical Sciences, Mashhad, Iran; e Assistant Professor of Anaesthetics, Mashhad University of Medical Sciences, Mashhad, Iran; f Resident of General Surgery, Mashhad University of Medical Sciences, Mashhad, Iran

**Keywords:** Leiomyomas, Bronchial Neoplasm, Bronchoscopic Surgical Procedure, Tracheal Incision, Immunolabeling Techniques

## Abstract

Bronchial leiomyoma is extremely rare. To date less than 60 cases have been reported in the world literature. The presented case here is a 30 year old woman who had been treated for bronchial asthma for several years. Her chest radiograph showed bulluos emphysematous changes in the right lung and computer tomography scan found the tumor in the right main bronchus near carina. Diagnosis was made by histological and immunohistochemical examination of the specimens obtained during bronchoscopy. The patient was treated by bonchoscopic resection of the 3 centimetre firm tumor and its removal through a tracheostomy incision.

Primary pulmonary leiomyomas are extremely uncommon both in adults and children, constituting approximately 2% of benign lung tumors.^1^ The affected patients usually have respiratory symptoms due to partial or complete airway obstruction which may stimulate asthma^1^,^2^ or be complicated with bronchiectasis and recurrent pulmonary infection.^1^,^3^,^4^

Herein an additional case with endobronchial leiomyoma is reported and a simple surgical method for removing large tracheal and bronchial lesions is explained. The literature is also reviewed.

## Case Report

A 30 year old non- smoker woman was admitted to our centre with a 10 year history of asthma like symptoms which did not respond to bronchodilators anymore.

**Figure 1:**
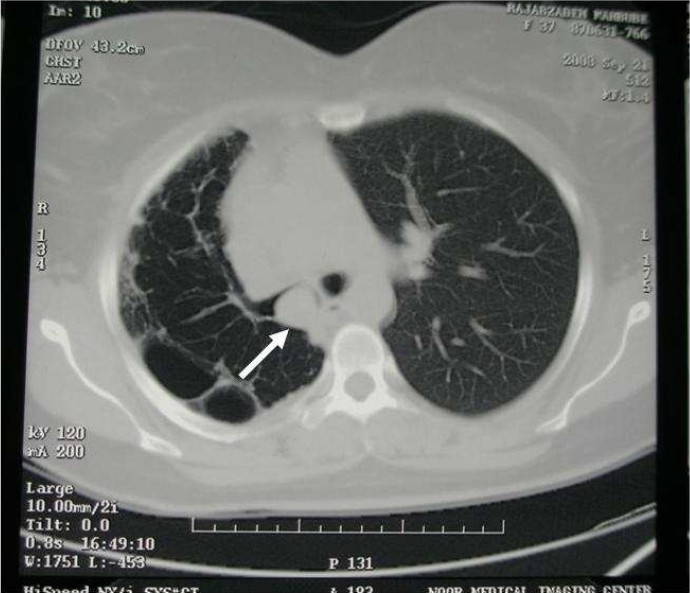
The tumor in proximal right main bronchus (RMB) in chest CT scan

Her chest radiograph showed bullous lesions, widespread infiltrates and volume reduction in the right lung. Computed tomography of the thorax demonstrated a mass lesion in the right main bronchus extending to the trachea as well as unilateral bullous emphysema with relatively thick septa, pleural thickness and reduced volume of the right hemi thorax (Figure 1).

**Figure 2:**
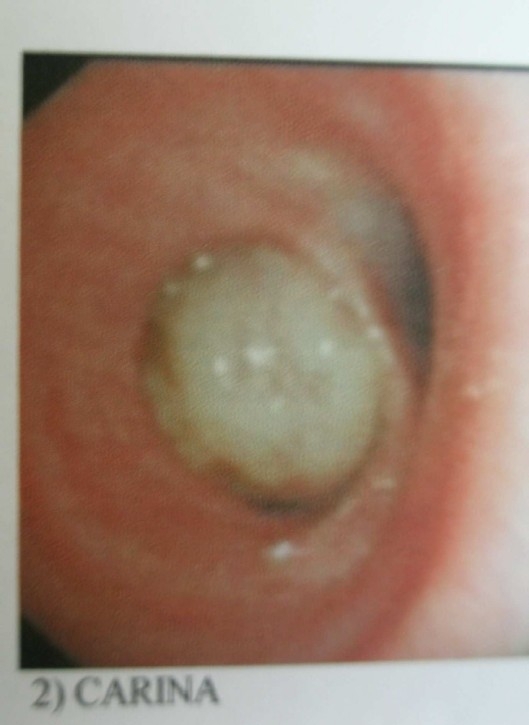
Fiber optic bronchoscopy shows the tumor in the opening of right main bronchus (RMB) near carina

Fiberoptic bronchoscopy detected a pink large mass completely obstructing the lumen of right main bronchus and extending to a point close to carina (Figure 2). Due to vascularised appearance of the mass and presumptive diagnosis of carcinoid tumor, biopsy was not attempted. Rigid bronchoscopy was performed under general anaesthesia and biopsy specimens were obtained.

**Figure 3:**
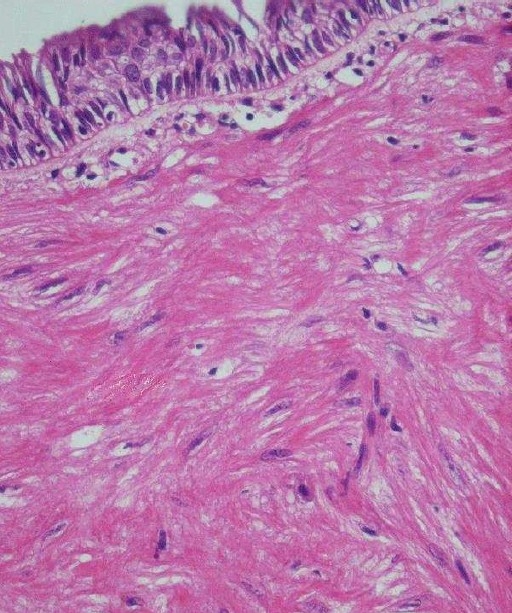
Histologic view of the tumor showing respiratory epithelium and subepithelial smooth muscle cell tumor (H&E staining 100 ×)

**Figure 4:**
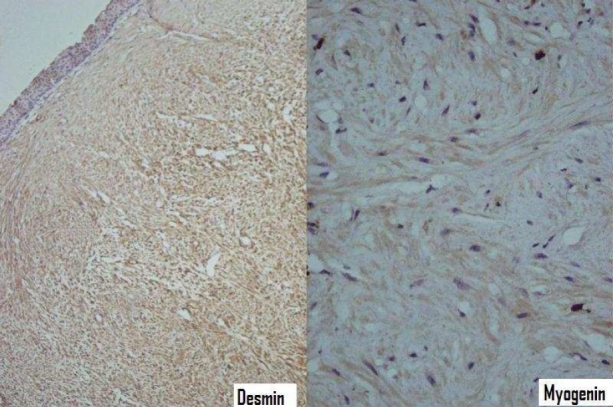
Immunohistochemical staining showing diffuse reactivity for desmin and negative myogenin reactivity (IHC staining 100 x, 400 ×)

Histopathologic examination revealed that immediately beneath the intact pseudostratified ciliated columnar epithelium with some focal squamous metaplesia, the neoplasm composed of bundles and whorls of spindle shaped cells with monomorphous fusiform nuclei and acidophilic cytoplasms without cytonuclear atypia, mitotic figures or necrosis (Figure 3). Also, sections of small blood vessels were observable. Immunohistochemically the tumor showed diffuse cytoplasmic reactivity for desmin while myogenin reactivity was negative (Figure 4). Ultrasonography of the uterus did not find any abnormality. On the basis of morphological and immunohistochemical findings the lesion was diagnosed as bronchial leiomyoma.

**Figure 5:**
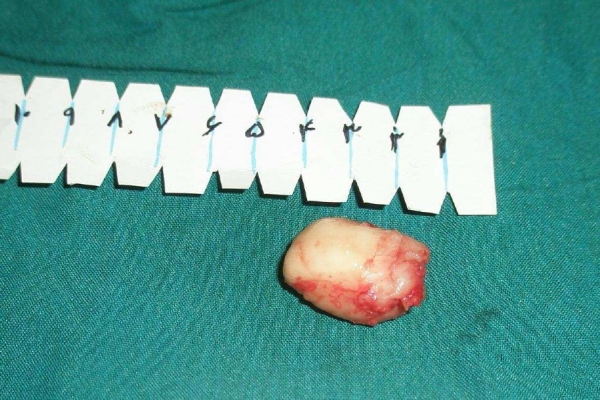
The tumor removed through tracheal incision after bronchoscopic resection

The patient was treated by endoscopic resection. However, the tumor was firm and large (3 centimeters in diameter) to remove through the rigid broncoscope despite great effort. In order to avoid tracheotomy and resection of a large portion of lung, a small cut was made in the neck area and the trachea was incised horizontally (tracheostomy). With cooperation of the anesthetics the tumor was brought to the incised region and removed by surgical forceps (Figure 5). Then the incisions were repaired. The patient was discharged after receiving medical treatment for her lung infection and showed remarkable improvement both clinically and on her control chest radiograph which was performed two weeks after surgery.

## Discussion

Primary leiomyoma is one of the most rarely encountered benign tumors of lung, accounting for less than 2% of all cases.^1^,^5^,^6^ These neoplasms can occur in parenchymal, endotreacheal or endobronchial locations. Endo- bronchial lesions constitute approximately 33% of all pulmonary leiomyomas.^5^ Generally it seems that pulmonary leiomyomas affect females more than males with a ratio of approximately 1.5:1^2^,^7^,^8^ ; however there is no gender predilection for endobronchial located leiomyomas.^5^ Although a wide age range, that includes pediatric cases, has been reported, most of the tumors tend to occur in individuals in the third and fourth decades of life^7^,^9^,^10^ as the presented case who was a female patient in her forth decade.

Symptoms depend on the location of the tumor, its size and the secondary changes of the lung distal to it. Patients with bronchial leiomyomas can have respiratory symptoms such as coughing, wheezing, dyspnea, chest pain or fever, due to partial or complete obstruction of the affected bronchus and superimposing infection resulting from atelectasis or bronchiectasis distal to obstruction).^1^–^4^,^11^ Intermittent or constant dyspnea and wheezing are the most common symptoms of tracheal leiomyoma and have been erroneously ascribed to bronchial asthma.^1^,^12^ In a case of leiomyoma in an accessory bronchus an emergency operation was performed due to severe dyspnea.^12^ The duration of asthma, like symptoms, before correct diagnosis has been as long as nine years.^13^ Parenchymal or peripheral leiomyoma are usually asymptomatic due to absence of symptoms caused by obstruction of the tracheo- bronchial tree.^8^,^9^ Clubbing of the fingers may be the only clinical sign in children while hemoptysis occur when the tumor surface ulcerates.^8^ The current case had asthma symptoms like coughing and dyspnea for nearly ten years and had repeatedly taken bronchodilators until the tumor obstructed the right main bronchus.

The chest radiographic findings of endo- bronchial leiomyomas range from normal in patients with small tumor nodule to a solitary round mass or pneumonic infiltration, mediastinal shift, and collapse of lung to unilateral emphysema or hyperlucency according to obstructive sequel of bronchus due to the tumor.^9^,^10^,^14^–^16^ A mass lesion with airway obstruction and/or pulmonary consolidation may be seen in computed tomograghy scans.^14^,^16^ The tumor most commonly manifested on CT scans as a homogeneously enhancing airway tumor with intraluminal growth. Also an iceberg appearance of the tumor (small intraluminal component and large extraluminal component) has been reported.^14^ Calcification may rarely occur in the lesion.^14^,^17^ In the present case, posteroanterior chest X- ray demonstrated pneumonic infiltration as well as bullous lesions and volume reduction of right lung. The CT findings showed that this bullous emphysema and volume reduction was caused by a soft tissue mass obstructing the right main bronchus extending near to carina.

The exact diagnosis of endobronchial leiomyoma can be made by broncoscopy in most cases because it can provide biopsy specimen for histological examination besides visualizing and localizing of the tumor.^4^,^7^ Tumors within the tracheobronchial tree appear as fleshy polypoid masses that protrude intralumenally and are attached to a wide base.^18^ In this case, a pink large mass completely obstructing the lumen of right main bronchus and extending to a point close to carina was detected by bronchoscopy, but the pulmonologist decided not to take biopsies because of the vascularised appearance of the mass that resembled a carcinoid tumor with a possible risk of bleeding; therefore it was performed by the thoracic surgeon under general anesthesia.

Bronchial leiomyomas are thought to derive from smooth muscle layer of bronchi, bronchiols, or blood vessels.^9^ Histological examinations of parenchymal pulmonary leiomyomas show that they are consisted largely of smooth muscle fibers, although an appreciable fibrous and vascular component is usually present. Bronchial leiomyomas are very cellular neoplasms, with minimal vascular or stromal fibrous component in contrast with parenchymal leiomyomas.^10^ Histological criteria, including cellularity, mitotic activity, necrosis and pleomorphism has been proposed to differentiate between benign and malignant smooth muscle neoplasms. The principle criterion is mitotic activity that should be less than 5 per 50 HPF.^19^ In the present case all morphologic findings were compatible with a benign spindle cell tumor.

Pulmonary nodules with features of small muscle tumors may be in fact metastasis from extra pulmonary leiomyoma/leiomyosarcoma. Uterine is the major primary site in such cases in females and sometimes the diagnosis of secondary pulmonary leiomyomatosis is made several years after hysterectomy.^20^,^21^ Ultra- sonography revealed no uterine neoplasm in the present case. Also, intrabronchial location, multiplicity of the lesion and admixed entrapped epithelial elements on histological examination can help to differentiate between metastatic smooth muscle tumors and their pulmonary counterparts.^7^,^20^,^21^

Differential diagnosis includes other spindle cell tumors and tumor lesions such as bronchial carcinoid of the large spindle cell variant, pleural fibrous mesothelioma, metastatic malignant melanoma, metastatic synovial sarcoma and occasionally plasma cell granuloma.^8^,^19^ To discriminate these lesions, immunohistochemical studies can be of great help in which both benign and malignant smooth muscle neoplasms show muscle markers like actin and desmim.^19^ Immunohistochemical staining for desmin and myogenin was performed here to find the exact nature of the tumor which confirmed the diagnosis of leiomyoma.

Leiomyomas of the respiratory system are essentially treated with surgical or bronchoscopic resection.^15^,^22^ The type of operation depends on the location of tumor and the presence of secondary lung destruction.^3^,^15^ Parenchymal liomyomas usually require a less radical procedure.^22^ On the other hand, bronchial leiomyomas have been treated by lobectomy, pnumonectomy, or segmentegtomy in cases with secondary parenchymal destruction,^8^,^9^,^22^ or more conservatively via different procedures such as bronchoscopic removal,^22^–^25^ excision with thracheostomy,^1^ sleeve resection of the involved bronchus and end to end anastomosis,^3^,^26^ and bronchotomy and bronchoplasty.^22^ Bronchoscopic intervention is a safe and effective technique for the treatment of patients with a tracheobronchial leiomyoma.^26^ In recent years the improving techniques of therapeutic bronchoscopy, besides being safe and effective, has been replacing the conventional surgery even in cases with complete bronchial obstruction.^23^,^24^,^27^ In general, bronchoscopic removal may be complicated by hemorrhage or perforation.^22^ Applying YAG laser or elecrocoatery may reduce the incidence of hemorrhage.^28^,^29^ In the current case, bronchoscopical resection was performed successfully but as the tumor was large in size and impossible to remove through a rigid broncoscope, it was removed through a tracheostomy incision with help of the anesthetics.

Development of smooth muscle neoplasm have reported in different conditions with immune deficiency, including acquired immunodeficiency syndrome (AIDS), particularly in children,^11^,^30^–^32^ cellular immune deficiency^33^ and malignant lymphoma.^34^ Epstein- Barr virus has also been detected in almost all AIDS- related smooth muscle neoplasms^30^,^35^,^36^ and in the reported case of bronchial leiomyoma in a boy with cellular immunodeficiency.^33^

## Conclusions

In summary, endobronchial leiomyomas are among the rarest of benign tumors of the respiratory tract. These neoplasms may mimic asthma or other obstructive diseases of the lung and should be kept in mind as a rare cause of asthma like symptoms especially in an adult female who is no longer responsive to bronchodilators. Histological examination and immunohistichemistry provide the exact diagnosis which can always exclude the possibility of a metastatic leiomyoma/leiomyosarcoma. Treatment is as conservative as possible and includes bronchial resection, segmentectomy, lobectomy or pnemonectomy.
